# Left-behind experience and language proficiency predict narrative abilities in the home language of Kam-speaking minority children in China

**DOI:** 10.3389/fpsyg.2022.1059895

**Published:** 2023-01-17

**Authors:** Wenchun Yang, Angel Chan, Natalia Gagarina

**Affiliations:** ^1^School of Foreign Studies, Xi’an Jiaotong University, Xi’an, China; ^2^Leibniz-Centre General Linguistics (ZAS), Berlin, Germany; ^3^Department of Chinese and Bilingual Studies, The Hong Kong Polytechnic University, Kowloon, Hong Kong SAR, China; ^4^Research Centre for Language, Cognition, and Neuroscience, The Hong Kong Polytechnic University, Kowloon, Hong Kong SAR, China; ^5^The Hong Kong Polytechnic University – Peking University Research Centre on Chinese Linguistics, Hong Kong, Hong Kong SAR, China

**Keywords:** narrative abilities, Kam-speaking, left-behind experience, linguistic proficiency, home language

## Abstract

**Introduction:**

Studies have documented that child experiences such as external/environmental factors as well as internal factors jointly affect acquisition outcomes in child language. Thus far, the findings have been heavily skewed toward Indo-European languages and children in the Western, educated, industrialized, rich and democratic (WEIRD) societies. By contrast, this study features an understudied minority language Kam, and a group of so-called left-behind children in China growing up in a unique social-communicative environment.

**Methods:**

Fifty-five bilingual children aged 5–9 acquiring Kam as home language were assessed using the Multilingual Assessment Instrument for Narratives (LITMUS MAIN). Twenty-three “two parents-left” children (mean age = 6;8, range: 5;0–9;2) remained in rural areas while both parents went to cities for employment, and they were raised by their grandparents. Thirty-two were “one parent-left” peers (mean age = 7;3, range: 5;0–9;3) who also resided in rural areas but were raised by one parent. Oral narrative texts were analysed for macrostructure based on story structure (SS), story complexity (SC) and internal state terms (IS). The study examined whether and how narrative production is predicted by internal factors such as chronological age and linguistic proficiency of a child and an external factor such as left-behind experience. Four measures were scored as outcome measures: SS, SC, IS type, IS token. Four measures were taken as predictors: chronological age, left-behind experience, scores in a lexical production task, and scores in a sentence repetition task tapping expressive morphosyntactic competence.

**Results:**

Results showed that left-behind experience consistently predicted all four outcome measures, where the “two parents-left” children scored significantly lower than their “one parent-left” peers. Expressive vocabulary scores predicted three measures: SS, SC, and IS Token. Expressive morphosyntactic scores predicted SS and SC. Age, by contrast, did not predict any outcome measure.

**Discussion:**

These findings suggested that being left-behind by both parents may be a negative prognostic indicator for the development and maintenance of heritage language abilities in ethnic minority children. We further discussed the conceptual significance of what it means for a child to be left-behind, by relating to more basic external factors in language development, including caregiver educational level, and amount of home language and literacy support by the caretakers.

## Introduction

Child experiences as well as internal factors jointly affect acquisition outcomes in child language (Paradis, 2011). While it is encouraging to note that there are increasingly more acquisition studies addressing internal and external factors in Asia (see, e.g., Dixon et al., 2012; Sun et al., 2016, 2018, 2020, 2022), the child language literature, however, is still heavily skewed toward Indo-European languages and the Western, Educated, Industrialized, Rich, and Democratic (WEIRD) societies (Henrich et al., 2010; Kidd and Garcia, 2022). This study, by contrast, features an understudied language, Kam, in a group of so-called left-behind children in China. Kam belongs to the Kam-Shui language branch of the Kam-Tai family and is spoken by 3.5 million minority Kam people residing in South (West) China (Office of Leading Group of the State Council for the Seventh National Population Census, 2021). Kam is a SVO language and has a complex and conservative tone system with up to 15 phonetic tones (Wu, 2018). Kam is undergoing language change due to intensive language contact with Chinese from formal schooling, TV broadcasting and employment (Yang, 2017). Kam does not have a widely used orthography or writing system but is transmitted more as an oral language. Kam-speaking people read and write in Chinese.

For decades as China continues its socioeconomic reform and urbanization, a large number of adult rural residents have been migrating to cities to seek work opportunities. Their young children, whose number exceeded 40 million by 2015 (National Bureau of Statistics of China, UNICEF China, UNFPA China, 2017), however, stayed behind in the rural areas due to various reasons. These children are called left-behind children (The State Council of the People’s Republic of China, 2016). There are two scenarios of the left-behind experience in terms of the number of parent(s) absent. One being the child staying with one parent (usually the mother) while the other parent goes to urban areas for work (one parent-left). Another being the child staying with the grandparents or other relatives while both parents leave for urban cities (two parents-left; Lu, 2012). In most cases, these caretakers are low-educated and lack the knowledge to take adequate care of these children and support language, psychological, cognitive, and other important aspects of child development. There are often a lack of quality and stimulating interactions between these children and caregivers.

Moreover, these children may experience generally reduced home language input. This is because most left-behind children grow up in low socioeconomic status (SES) families and parents from low SES families often encourage their children to use the majority language more often than their home (i.e., minority) language to become more successful at school (Lambert and Taylor, 1996). This parental preference could lead to further challenges in these children’s home language development and maintenance. Furthermore, most left-behind children live in remote rural areas in poor provinces in Western and Southwestern China where there is limited access to resources and facilities for learning such as books and libraries (Han et al., 2017). Taken together, the prolonged absence of parental care, the loss of solid family structure, poor living conditions and lack of learning resources make these children more vulnerable to developmental, behavioral and psychological problems (Wen and Lin, 2012; Wang and Mesman, 2015; Lu et al., 2021).

These left-behind children in general and their language development, in particular, have not received much attention in the developmental literature. While there are differences in reasoning abilities (Liu et al., 2018) and social skills (Hu et al., 2020) between the one parent-left and two parents-left groups, the impact of this unique social-communicative environment on language development of these left-behind children, especially their home language development, awaits further investigation.

In this paper, we focus on children’s narrative abilities, as narratives are an indispensable part of one’s social life. Narrative competence is also a strong predictor of children’s later academic achievement in literacy, reading and mathematics (e.g., Hayward and Schneider, 2000; O’Neill et al., 2004; Swanson et al., 2005; Oakhill and Cain, 2012). Narrative abilities can be evaluated on macrostructure and microstructure levels. Macrostructure is the global setting of a story, referring to a higher order of hierarchical organization of episodes and story grammar components such as characters’ goal, attempt, outcome and reaction (Heilmann et al., 2010), and draws upon cognitive skills and theory of mind abilities. Macrostructure includes three components: Story structure (SS), story complexity (SC) and internal state terms (IS) (Gagarina, 2016). SS captures the quantitative dimension by counting the number of story grammar elements expressed, e.g., Setting, Initiating Event, Goal, Attempt, Outcome, and Reaction. SC captures the qualitative dimension by examining children’s ability to combine the main episodic elements Goal-Attempt-Outcome to verbalize a complete episode in narratives. IS are words denoting mental states including, for instance, perceptual state (e.g., see, hear), physiological state (e.g., thirsty, hungry), consciousness (e.g., alive, awake), emotion (e.g., sad, happy), and mental verbs (e.g., want, think, know).

Macrostructure has been shown to be affected by multiple factors, both external and internal (Gagarina, 2016). The current study targets the higher-order organization of narratives. It therefore addresses macrostructure and contributes to the existing research by reporting data on narrative production by Kam-speaking children in their home language, examining the role of internal and external factors on narrative organization. External factors relate to children’s language environment and experiences including parental education and language use. Internal factors refer to those related to children’s inherent characteristics including, for instance, chronological age (henceforth age), IQ, linguistic proficiency (Armon-Lotem et al., 2011). In the first round of analyses, we focused on one external factor (children’s left-behind experience), and two internal factors (chronological age, linguistic proficiency) which have been reported to be associated with children’s narrative macrostructure production (Lindgren and Bohnacker, 2021). In the subsequent analyses, we further investigated children’s left-behind experience by examining child caretaker characteristics and the quality of home experiences/environment and degree of family support and engagement. The following section elaborates on the relevant literature.

## Factors influencing children’s narrative macrostructure production

### Left-behind experience

To our knowledge, no studies have investigated the effect of left-behind experience on narrative macrostructure production. There are, on the other hand, two studies examining the relationship between left-behind experience and children’s cognitive skills and receptive vocabulary competence. A longitudinal study by Hu et al. (2020) reported that left-behind preschoolers who stayed with one parent performed better in executive functioning tasks and Chinese reading than those who stayed with their grandparents with both parents being absent. Ding et al. (2021) reported that left-behind children aged 4–6 staying with one parent scored higher in receptive vocabulary than their peers who stayed with grandparents. Similarly, Liu et al. (2018) reported that the development of theory of mind in school-aged left-behind children was slower than the non-left-behind children. Overall, left-behind children, especially whose parents were both absent, scored lower in these cognitive and language tasks.

Left-behind experience bears on the quantity and quality of home experiences/environment and degree of family support and engagement, which have been shown to highly correlate with children’s development of narrative abilities. For example, in the longitudinal study on Spanish-English bilinguals with low-income backgrounds from preschool to first grade, Bitetti and Hammer (2016) reported a positive impact of home language experience on children’s English narrative macrostructure skills. Being frequently exposed to literacy activities (e.g., book reading) allows children to internalize the global structure of narrative and use it when they tell their own stories. Relating to these left-behind children, long-term family separation causes challenges including limited quality parent–child communication and limited home literacy-related activities such as shared book reading and storytelling, which are not conducive to children’s cognitive and linguistic development.

### Linguistic proficiency

Linguistic proficiency has been reported to be a significant predictor of narrative macrostructure (e.g., Lindgren, 2018; Fiani et al., 2021). Conceptually this relationship is reasonable as narrative macrostructure production requires support from foundational linguistic skills including lexical and morphosyntactic competence. Children need to use diverse and appropriate vocabulary, syntactic structure and morphology to formulate a story. A number of studies have shown that expressive vocabulary is significantly associated with children’s narrative macrostructure skills (e.g., Uccelli and Páez, 2007; Lindgren and Bohnacker, 2020, 2021). Uccelli and Páez (2007) reported a significant correlation between expressive vocabulary and story structure in 5–7 years old Spanish-English bilinguals in their two languages. Interestingly, Lindgren and Bohnacker (2021) elicited story narratives from a group of 4–6 years old German-Swedish bilinguals and reported that expressive vocabulary predicted children’s story structure performance only for German (the minority language) but not for Swedish (the majority language). The authors reasoned that children need to achieve a certain level of lexical skills to narrate a story with a well-formed global organization. Some German-Swedish children might not have achieved this “threshold” level of vocabulary competence in their weaker minority language German and therefore their weak lexical skills could restrict their expressive narrative macrostructure. If so, the association between lexical skills and expressive narrative macrostructure could be tighter/stronger in the weaker minority language than the majority language.

The relationship between morphosyntactic skills and macrostructure was less examined in previous studies. Some studies reported positive correlations between narrative macrostructure and morphosyntactic competence as reflected by children’s narrative microstructure skills. For instance, Iluz-Cohen and Walters (2012) examined narrative production in English-Hebrew preschoolers and reported that story structure scores correlated with children’s narrative microstructure skills in morphosyntax in both languages. Rodina (2017) examined a group of Norwegian-Russian bilingual children aged 4;6 and reported that expressive narrative macrostructure scores correlated with their mean length of utterance, a measure of morphosyntactic competence, in children’s home minority language Russian. These studies, however, derived their measures on narrative macrostructure competence and morphosyntactic competence from the same narrative samples. Further research can use independent measures of morphosyntactic competence to further evaluate the relationship between narrative macrostructural competence and morphosyntactic competence in children’s home language.

### Age

Age has also been reported as a significant predictor of children’s narrative abilities (Bohnacker, 2016; Maviş et al., 2016; Roch et al., 2016; Lindgren and Bohnacker, 2021). In general, older children score higher in story structure and produce a higher level of story complexity as they are supported by more advanced cognitive and linguistic skills to express the contents and temporal-causal relationships in a story. In addition, older children may also have more opportunities to take part in literacy-related activities such as book reading and storytelling, which help them acquire more skills and knowledge to organize a story. On the other hand, it is possible that age effects are less prominent in a minority home language acquisition context, when language outcome measures are more affected by environmental factors such as amount of target language exposure and home literacy-related activities (Bohnacker et al., 2021; Lindgren and Bohnacker, 2021). Bohnacker et al. (2021) studied the age effect on narrative macrostructure elicited from 100 Turkish-Swedish bilingual children aged 4–7. They reported a weaker relationship between age and children’s story structure scores in the home language Turkish. The same pattern was reported by Lindgren and Bohnacker (2021) who reported that the age effect on macrostructure performance was weaker in the home language, German, of forty-one German-Swedish bilinguals aged 4–6. Age effects were weaker in these children’s macrostructure competence in their home minority language, likely because home minority language is often associated with lower exposure to literacy-related activities (Bitetti and Hammer, 2016).

## The current study

This study examines whether and how the external factor [i.e., left-behind experience (one parent-left and two parents-left)] and internal factors (i.e., linguistic proficiency measured by lexical and morphosyntactic skills, and age) affect children’s expressive narrative macrostructure in their home minority language. Narrative macrostructure is operationalized as SS, SC and IS tokens and types (see above for more details). Each research question addresses one macrostructure component/dimension. The research questions and their predictions are stated below:

1. Does left-behind experience predict the production of narrative macrostructure in the minority language Kam?

Prediction: Since left-behind experience has been reported to negatively correlate with the development of cognitive and linguistic skills (Liu et al., 2018; Hu et al., 2020; Ding et al., 2021), and that these foundational abilities support narrative competence, it is reasonable to expect that the outcome measures in narrative macrostructure would be significantly affected by this factor. Specifically, children who are left behind by both parents are expected to score lower in macrostructure than those who are left behind by only one parent.

2. Does linguistic proficiency predict narrative macrostructure in Kam?

Prediction: Consider that linguistic proficiency indexed by lexical and morphosyntactic competence has been reported as a significant predictor of children’s narrative abilities (Bohnacker et al., 2021; Fiani et al., 2021), and that the effect is stronger in the home minority language (Lindgren and Bohnacker, 2021), we predicted a strong association between vocabulary and morphosyntactic abilities and narrative abilities in Kam.

3. Does age predict narrative macrostructure in Kam?

Prediction: Consider studies which have reported weaker age effects in a minority home language acquisition context, when language outcome measures are more affected by environmental factors such as amount of target language exposure and home literacy-related activities (Bohnacker et al., 2021; Lindgren and Bohnacker, 2021), as well as our expectation that the home language environment of these left-behind children is often associated with insufficient language learning support and resources and literacy-related activities in the home language, we predict a weak or even no significant age effect on narrative macrostructure.

## Materials and methods

### Participants

Fifty-five (*N* = 55) Kam-Mandarin ethnic minority bilingual children aged 5 to 9 participated in this study with written consent from their caretakers. Twenty-three (*N* = 23) were two parents-left children (two parents-left group; mean age = 6;8, range: 5;0–9;2) who remained in rural areas while their parents both went to cities for employment and were raised by their low SES caretakers. Thirty-two (*N* = 32) were SES matched one parent-left peers (one parent-left group; mean age = 7;3, range: 5;0–9;3) who also resided in rural areas but only one parent went to cities for work. All participants acquired Kam as home and first language (L1) and Mandarin as school and second language (L2) from age 3. All children were recruited from Guangxi Zhuang Autonomous Region in South China and lived in a town with the majority of the Kam population speaking Kam from birth and had never lived in another place for more than 1 month. All children attended kindergarten (5–7 h a day) and primary school (7–8 h a day) with formal education in L2 Mandarin. According to the care-taker questionnaire (Gagarina et al., 2019), these children had no reported learning disabilities and neurological, psychological, or social disorders.

### Materials

#### Linguistic proficiency

Children’s linguistic proficiency was assessed in terms of expressive lexical ability and morphosyntactic ability. Children’s expressive lexical competence was assessed by the Multilingual Naming Test (MINT, Gollan et al., 2012; Ivanova et al., 2013). Children were required to name the object depicted in the picture. A score of 0 or 1 was assigned to each picture according to accuracy of response (0-incorrect, 1-correct). The full score is 67 marks. Children’s morphosyntactic competence was assessed by a sentence repetition task (SRep) adapted into Kam (Marinis and Armon-Lotem, 2015). SRep consists of SVO sentences with auxiliaries, negation, aspect marker, biclausal complement (e.g., “After Father had dinner in the evening, he went to take a shower.”) and complex sentences including wh-questions, relative clauses, and passives. There are 57 sentences in total and each sentence contains 9–13 syllables. Children’s responses were scored 0 (incorrect) or 1(correct). A score of 1 was given to only responses which were exactly the same as the target structures.

#### Narrative production

Children’s narrative production abilities were assessed by the Kam version of the Multilingual Assessment Instrument for Narratives (LITMUS MAIN, Gagarina et al., 2015, 2019; Kan et al., 2020; Yang et al., 2020). MAIN is an assessment tool for narrative skills which has been adapted into 92 language versions and is widely used in testing children’s story narrative competence cross-linguistically. It consists of four picture-based stories: Cat, Dog, Baby Birds and Baby Goat. Each story has six pictures consisting of three episodes. All four stories were used to elicit narratives.

We followed the standard guidelines of MAIN in task administration (Gagarina et al., 2019). Children first looked at the pictures and then were asked to tell and retell the relevant stories in Kam. Stories for retelling were pre-recorded by a native speaker. Children’s narrated stories were transcribed verbatim by a trained native speaker. The data were coded following the scoring form of MAIN. 20% data were transcribed and coded by a second trained native speaker for intercoder reliability check. The percentage of agreement in transcription and coding was 99.0 and 97.0%, respectively.

Three components of macrostructure were evaluated: SS, SC, and IS. SS has a maximum score of 17. This score is derived from the five story grammar elements of an episode, IS as Initiating Event, Goal, Attempt, Outcome, IS as a Reaction (one mark for one element), multiplied by the number of episodes (3) in a story, with 2 more points given for the story setting (time and place). SC has a maximum score of 3 for each episode. A score of 0 was given if neither G, A nor O was expressed in an episode. A score of 1 would be given to a sequence without G (i.e., A, O or a combination of AO), a score of 2 was given to an incomplete episode with G, or a combination of GA or GO. A complete episode with GAO all verbalized was given 3 marks. As for IS, both token and type measures were scored (1 token/type, 1 score).

#### Home language environment

As we will see in section “Further analyses” we will further discuss what it means for a child to be left-behind, by relating to more basic external factors in language development including amount of home language use by caretakers, home literacy support, and education level of caretaker(s). To address this, we refer to data collected by a caregiver questionnaire (Gagarina et al., 2019). The questionnaire asks questions about children’s language background, left-behind experience, caregiver’s amount of home language use and education level (in terms of years of education), and home literacy support indexed by frequency of storytelling at home (e.g., “How often do you do storytelling with your child in the last month?”) and number of non-textbooks the child has at home (e.g., “How many non-textbooks do you have at home?”). The number of non-textbooks at home was reported on a 5-point scale: 0 = 0–5 books; 1 = 5–20 books; 2 = −20–50 books; 3 = 50–100 books; 4 = more than 100 books. The caregiver rated the frequency of storytelling on a 4-point scale: 0 = never, 1 = twice a month, 2 = once or twice a week and 3 = almost every day. Amount of home language use by caregiver(s) in response to the question “How much Kam do you use in your daily communication with your child?” was also rated on a 5-point scale: 0 = never, 1 = seldom, 2 = sometimes, 3 = usually, 4 = always. Caretakers’ education level was calculated in terms of years of education completion.

## Results

### First round of analyses

#### Descriptive statistics

Table 1 shows the descriptive statistics of children’s scores in the four narrative outcome measures (i.e., SS, SC, IS type and token). The two parents-left group scored numerically lower than the one parent-left group across all measures of narrative macrostructure. Mann–Whitney U test showed significant group differences in SS (*p* < 0.05) and IS type (*p* < 0.01). Despite no significant group difference in SC (*p* > 0.05), qualitative analyses showed that there were fewer children in the two parents-left group who could produce at least one complete GAO episode, relative to the one parent-left group [56.52% (13/23) vs. 68.75% (22/32)].

**Table 1:** Descriptive statistics of children’s scores in each outcome measure of macrostructure competence (mean (SD) and score range).

Left-behind experience	Story structure (SS)		Story complexity (SC)		Internal state terms (types)		Internal state terms (tokens)	
	Mean (SD)	Range	Mean (SD)	Range	Mean (SD)	Range	Mean (SD)	Range
Two parents-left	6.22 (2.57)	2–14	4.43 (1.40)	2–9	2.74 (0.54)	1–4	4.74 (1.84)	2–12
One parent-left	7.80 (2.34)	1–15	5.04 (1.25)	2–9	3.22 (0.66)	2–4	5.59 (1.48)	3–10

#### Effects of left-behind experience, linguistic proficiency, and age

Correlations between predictors in the two rounds of analyses were first computed. Weak and moderate correlations (i.e., correlation coefficients below 0.7; Ratner, 2009) were found (Table 2), signaling a low degree of multicollinearity.

**Table 2:** Summary of intercorrelations between predictors.

	1	2	3	4	5	6	7
1. Left-behind experience	1						
2. Lexical competence	0.071	1					
3. Morphosyntactic competence	0.075	0.378***	1				
4. Caregiver’s education level	−0.558***	0.001	0.120	1			
5. No. of non-textbooks	−0.213*	−0.057	0.051	0.469***	1		
6. Frequency of storytelling in Kam	−0.132	−0.344***	−0.003	0.152	−0.043	1	
7. Amount of Kam use	0.161	0.139	0.170	−0.221*	−0.159	−0.152	1

^*^p < 0.05, ^**^p < 0.01, ^***^p < 0.001.

A linear mixed-effects model was run in R (version 4.2.0; R Core Team, 2022) with the lme4 package (version 1.1–18-1, Bates et al., 2015). Left-behind experience (one parent-left vs. two parents-left), expressive vocabulary scores, expressive morphosyntax scores and age were included as fixed effects, and participants as a random effect. A top-down model building strategy was adopted by starting with a full model and stepwise removing predictors that did not significantly contribute to the model fit. The model fit was tested by comparing the two subsequent models using the *anova* function.

Table 3 presents the significant terms in the final model for each narrative outcome measure. SS scores were negatively predicted by left-behind experience (*β* = −1.936, SE = 0.486, *t* = −3.980, *p* < 0.001) and positively predicted by lexical (*β* = 0.085, SE = 0.024 *t* = 3.521, *p* < 0.001) and morphosyntactic competence (*β* = 0.156, SE = 0.035, *t* = 4.517, *p* < 0.001). SC scores were negatively predicted by left-behind experience (*β* = −0.740, SE = 0.306, *t* = −2.420, *p* < 0.05) and positively predicted by both lexical (*β* = 0.046, SE = 0.015, *t* = 3.032, *p* < 0.01) and morphosyntactic competence (*β* = 0.051, SE = 0.021, *t* = 2.329, *p* < 0.05). IS scores (type measures) were negatively predicted by left-behind experience (*β* = −0.463, SE = 0.159, *t* = −2.906, *p* < 0.01) and not other factors. IS scores (token measures) were negatively predicted by left-behind experience (*β* = −0.954, SE = 0.426, *t* = −2.236, *p* < 0.05) and positively predicted by lexical competence (*β* = 0.054, SE = 0.019, *t* = 2.754, *p* < 0.01).

**Table 3:** Significant terms in the final model for performance in each outcome measure of macrostructure competence in the first round of analyses.

Measure	Predictor	Estimate	SE	*t*
Story structure	(Intercept)	−2.643	1.534	−1.723
	Left-behind experience	−1.936	0.486	−3.980***
	Lexical competence	0.085	0.024	3.521***
	Morphosyntactic competence	0.156	0.035	4.517***
Story complexity	(Intercept)	0.901	0.966	0.933
	Left-behind experience	−0.740	0.306	−2.420*
	Lexical competence	0.046	0.015	3.032**
	Morphosyntactic competence	0.051	0.021	2.329*
Internal state terms (types)	(Intercept)	2.984	0.103	28.984***
	Left-behind experience	−0.463	0.159	−2.906**
Internal sate terms (tokens)	(Intercept)	3.440	0.834	4.123***
	Left-behind experience	−0.954	0.426	−2.236*
	Lexical competence	0.054	0.019	2.754**

^*^p < 0.05, ^**^p < 0.01, ^***^p < 0.001.

Taken together, left-behind experience negatively predicted all four outcome measures in macrostructure competence, indicating that two parents-left children scored significantly lower than one parent-left children. Expressive lexical competence positively predicted SS, SC, and IS (tokens) scores. Morphosyntactic competence positively predicted SS and SC scores. Age did not contribute to the model fit and was removed from the model, indicating that age was a not a significant predictor for all outcome measures.

### Further analyses

Left-behind experience, a general notion, is associated with a number of characteristics impacting different facets of life, including amount of home language and literacy support that is important for a child’s language development. We therefore conducted some further analyses to examine whether/how some more basic external factors associated with left-behind experience might predict these narrative outcome measures (Bitetti and Hammer, 2016; Pace et al., 2017). Specifically, we examined the effect(s) of caregiver education level, amount of home literacy support indexed by the number of non-textbooks at home and frequency of storytelling in Kam at home, and amount of home language support indexed by amount of Kam the caregiver used with the child. Linear mixed-effects models were run with left-behind experience being replaced by these external factors, while keeping expressive lexical and morphosyntactic scores as fixed effects, and participants as random effects.

Results (Table 4) indicated that caregiver education level positively predicted all four outcome measures of macrostructure competence. Expressive lexical competence positively predicted SS (*β* = 0.084, SE = 0.024, *t* = 3.544, *p* < 0.001), SC (*β* = 0.056, SE = 0.014, *t* = 3.909, *p* < 0.001) and IS (tokens) scores (*β* = 0.050, SE = 0.019, *t* = 2.593, *p* < 0.05). Morphosyntactic scores positively predicted SS scores (*β* = 0.133, SE = 0.035, *t* = 3.827, *p* < 0.001) and no longer SC scores. Again, no significant age effects were registered. The number of non-textbooks at home and the frequency of storytelling at home were not significant predictors. This is likely due to the generally low numeral values of these variables with small variations within each variable, and therefore did not yield any significant results. Specifically, 95% of caretakers reported fewer than 5 non-textbooks at home and these children also seldom had storytelling activities at home. Interestingly, the amount of home language use by caregiver(s) was not a significant predictor either. We will discuss our speculation in the discussion section.

**Table 4:** Significant terms in the final model for performance in each outcome measure of macrostructure competence in the follow up analyses.

Measure	Predictor	Estimate	SE	*t*
Story structure	(Intercept)	−4.607	1.585	−2.906**
	Education	0.372	0.099	3.736***
	Lexical competence	0.084	0.024	3.544***
	Morphosyntactic competence	0.133	0.035	3.827***
Story complexity	(Intercept)	1.651	0.730	2.263*
	Education	0.135	0.065	2.076*
	Lexical competence	0.056	0.014	3.909***
Internal state terms (types)	(Intercept)	2.357	0.218	10.817
	Education	0.072	0.033	2.146*
Internal state terms (tokens)	(Intercept)	1.822	0.979	1.862
	Education	0.222	0.086	2.576*
	Lexical competence	0.050	0.019	2.593*

^*^p < 0.05, ^**^p < 0.01, ^***^p < 0.001.

## Discussion

We reported the first empirical study investigating left-behind Kam-speaking children’s narrative abilities and their predictors in their home language, Kam. Specifically, we examined whether the external factor indicated by left-behind experience, and internal factors indicated by lexical and morphosyntactic skills and age predict the expressive narrative macrostructure competence in a group of children aged 5–9. Left-behind children were divided into two groups depending on whether one or two parents left for urban areas. Since *left-behind* is a composite phenomenon, we further examined caregiver characteristics and amount of home language and literacy support by caregivers, including caregiver education level, amount of home literacy support indexed by number of non-textbooks at home and frequency of story-telling activities at home, and amount of home language use by the caregiver to the child. There were four outcome measures of macrostructure competence: SS, SC, IS (types) and IS (tokens). As expected, left-behind experience negatively predicted performance in all four outcome measures. Lexical competence positively predicted SS, SC and IS (tokens) scores. Morphosyntactic competence positively predicted SS and SC scores. No significant age effects were found. Below we discuss each predictor.

Left-behind experience as a *whole* negatively predicted narrative macrostructure competence, indicating that children who were raised by their grandparents/relatives scored lower than those who were raised by one of the parents across all four outcome measures. Further analyses showed that caregiver’s education level positively predicted all four outcome measures of macrostructure competence. More educated caregivers often can provide more learning support and stimulating adult-child communication that are conducive to child language development. In general, these children’s parents have higher education level than their grandparents/relatives. “One parent-left” children likely have more language learning support from their higher educated parent than the “two parents-left” children raised by lower educated grandparents/relatives. Home literacy support indexed by number of non-textbooks at home and amount of story telling activities at home, and home language support indexed by amount of home language use by the caregiver to the child did not turn out to be significant predictors of these outcome measures either. This is likely due to the generally low values and small variations within factor for these predictors (see Results section). Our speculation is that although Kam is the children’s home language, the caregivers seldom had communication with the children.

Expressive lexical competence positively predicted performance in SS, SC and IS (tokens) scores. This finding partially aligns with previous results. For instance, Bohnacker et al. (2021) and Lindgren and Bohnacker (2021) reported a positive relationship between expressive lexical competence and SS in Turkish-Swedish bilingual children and German-Swedish bilingual children, respectively. These two studies, however, did not examine the effect of lexical competence on SC and IS. Gagarina (2016) examined expressive macrostructure competence in a group of Russian-German bilingual children aged 4–9 and reported that performance on SS and SC, but not IS, was invariant between languages. Based on these findings, she suggested that SS and SC are less language dependent, whereas IS is more language dependent and contingent on language-specific lexical knowledge. The current finding, on the other hand, indicates that the three outcome measures (SS, SC and IS) are all dependent on lexical competence in the target language. This might be due to the restricted expressive vocabulary competence in Kam in these children. Children need a *critical mass* of lexical items in their repertoire in order to support them to express story grammar elements. Previous studies did not consistently register a significantly positive relationship between lexical and macrostructure competence, likely because some children in those studies exceeded the so-called “threshold” level of lexical competence, and therefore their macrostructural performance was less restricted/dependent on lexical competence scores (that is, children scoring lower or higher in lexical measures, would not be disadvantaged or advantaged in their narrative macrostructure competence, as both would still have adequate vocabulary to support expression of basic story grammar elements; see Gagarina et al., 2019; Lindgren and Bohnacker, 2021). IS, on the other hand, by nature depends on vocabulary size of the child. Morphosyntactic competence predicted SS and SC. This is conceptually reasonable as expression of story grammar elements requires foundational morphosyntactic abilities to combine words together (Iluz-Cohen and Walters, 2012; Lindgren and Bohnacker, 2021).

Age was not a significant predictor, as expected. This is consistent with previous results by Bohnacker et al. (2021) and Lindgren and Bohnacker (2021) which reported only a weak relationship between age and macrostructural performance in the home language. In our study, there was not even a weak age effect, and we suspected that this is due to the unique non-conducive socio-communicative environment of these left-behind children, causing the associated external factors such as left-behind experience and educational level of caregivers to be particularly prominent in their effects on these children’s narrative competence, rather than in a scenario where we would see age-related progress in narrative competence as a result of cumulative experience from a more conducive socio-communicative environment as children grow older.

## Conclusion

Although there are a growing number of studies examining the left-behind children in rural areas of China, very few studies have examined these children’s home language development. This study makes a first attempt to fill this gap by focusing on expressive narrative macrostructure abilities in their home language. We document that left-behind experience negatively predicted children’s narrative competence, and foundational lexical and morphosyntactic abilities positively predicted children’s narrative competence, while chronological age was not a significant predictor. Children growing up with both parents absent scored significantly lower than those growing up with one parent. More educated caregivers are associated with better narrative competence. This study has several limitations and these limitations should be considered in future research. First, the sample size is relatively small and future research should include more participants. Second, we had limited information regarding influence from other people whom children have immediate contact with, including teachers at school, classmates, playmates in the village, etc. Apart from the caretakers, these people also have potential influence on children’s language development. This information should be collected in future studies.

Our findings offer some important implications for policies and practices that are pertinent to this group of disadvantaged population. The strikingly low number of non-textbooks these children have at home and the low frequency of home literacy activities such as storytelling at home warrant public attention. We hope documenting these findings could be informative to parents, educators, and policymakers as they reflect on how they can better support the language development of these left-behind children. Moreover, our study is the first to investigate oral narratives in the under-investigated language Kam and thus might have important implication for language teaching and education in ethnic minorities in China. Last but not least, our results could contribute to preserving indigenous languages and cultures which are critical to making our the world more sustainable and diverse.

## Data availability statement

The raw data supporting the conclusions of this article will be made available by the authors, without undue reservation.

## Ethics statement

The studies involving human participants were reviewed and approved by the Human Subjects Ethics Sub-committee at the Hong Kong Polytechnic University (reference number: HSEARS20190916002). Written informed consent to participate in this study was provided by the participants' legal guardian/next of kin.

## Author contributions

WY, AC, and NG participated in the design of this study and interpretation of the data and drafted the manuscript and revised it critically and performed the final edits. WY was responsible for data collection, data coding, and data analyses. All authors contributed to the article and approved the submitted version.

## Funding

This study was supported by a research stipend by the Fritz Thyssen Foundation to WY (Ref. 40.20.0.002SL, PI: Yang).

## Conflict of interest

The authors declare that the research was conducted in the absence of any commercial or financial relationships that could be construed as a potential conflict of interest.

## Publisher’s note

All claims expressed in this article are solely those of the authors and do not necessarily represent those of their affiliated organizations, or those of the publisher, the editors and the reviewers. Any product that may be evaluated in this article, or claim that may be made by its manufacturer, is not guaranteed or endorsed by the publisher.
